# Characterization of invasive *Neisseria meningitidis* strains from Québec, Canada, during a period of increased serogroup B disease, 2009-2013: phenotyping and genotyping with special emphasis on the non-carbohydrate protein vaccine targets

**DOI:** 10.1186/s12866-015-0469-6

**Published:** 2015-07-25

**Authors:** Dennis K.S. Law, Brigitte Lefebvre, Rodica Gilca, Saul Deng, Jianwei Zhou, Philippe De Wals, Raymond S.W. Tsang

**Affiliations:** Vaccine Preventable Bacterial Diseases, National Microbiology Laboratory, Public Health Agency of Canada, 1015 Arlington Street, R3E 3R2 Winnipeg, MB Canada; Laboratoire de santé publique du Québec, Institut national de santé publique du Québec, 20045 chemin Sante-Marie, Ste-Anne-de-Bellevue, H9X 3R5 Québec, Canada; Institut national de santé publique du Québec, Centre de Recherche du CHUL-CHUQ, Québec, Canada; Département de Médecine Sociale et Préventive de I’Université Laval, Québec, Canada

**Keywords:** Invasive *Neisseria meningitidis*, 4CMenB protein vaccine targets

## Abstract

**Background:**

The epidemiology of invasive meningococcal disease (IMD) in Québec, Canada, has been dominated in the past decade by a clone of serogroup B (MenB) *Neisseria meningitidis* defined by multi-locus sequence typing (MLST) as sequence type (ST)-269. With the licensure of a new MenB vaccine Bexsero (4CMenB) in Canada, this study characterized invasive *N*. *meningitidis* recovered in Québec from 2009 to 2013, with an objective to examine the diversity of the 4CMenB vaccine antigens. Isolates were serogrouped by antisera and genogrouped by PCR, and further typed by whole cell ELISA for serotype and serosubtype antigens. Clonal analysis was done by MLST. Isolates were genotyped by analysis of their 4CMenB vaccine antigen genes of PorA, factor H binding protein (fHbp), Neisserial Heparin Binding Antigen (NHBA), and Neisseria Adhesin A (NadA).

**Results:**

Of the 263 IMD isolates analysed, 229, 16, 10, 7, and 1 belonged to MenB, MenY, MenW, MenC, and MenX, respectively. Of the 229 MenB, 159 (69.4 %) were typed as ST-269 clonal complex (CC); and they possessed a restricted number of three *fHbp* and five *nhba* gene alleles. Nine *N. meningitidis* isolates (eight MenB and one MenY) were found to possess at least one gene that encoded for an antigen that matched exactly with protein variants in the 4CMenB vaccine. Two MenB expressed PorA antigen P1.4 and possessed the *nhba* gene for peptide 2; four other MenB were predicted to have NHBA peptide 2; another two MenB were predicted to encode fHbp peptide 1.1; and a single MenY was found to have *nadA* gene for NadA peptide 8. In addition, another 172 isolates were found to possess genes for variant 1 fHbp peptides other than peptide 1.1 or NadA variant 1-2/3 peptides other than peptide 8; and therefore, may potentially be covered by 4CMenB.

**Conclusion:**

The most prevalent clone of *N. meningitidis* in Quebec was ST-269 CC; and 96 % of the isolates in this CC were predicted to be covered by 4CMenB vaccine. Extensive genetic diversity was found in the other IMD isolates in Québec which might suggest a lower coverage by the vaccine when compared to the ST-269 MenB.

**Electronic supplementary material:**

The online version of this article (doi:10.1186/s12866-015-0469-6) contains supplementary material, which is available to authorized users.

## Background

*Neisseria meningitidis* is a Gram-negative diplococcus which infects only humans. On average 10 % of healthy individuals may carry this bacterium in their nasopharynx, and due to reasons not completely understood, the bacterium sometimes gains access into the blood stream and causes systemic diseases such as meningitis, septicemia, and occasionally localised infections in the joint, and heart [1]. Six of the 12 known serogroups (A, B, C, W, X, and Y) of meningococci are responsible for most of the invasive diseases which occur worldwide [2, 3]. Although all 6 serogroups may cause outbreaks, serogroups A, B, C, and W have caused epidemics that may spread globally [4]. Case fatality rates associated with the different serogroups and in particular clonal groups may also vary [5, 6].

Besides the capsule, typing of *N. meningitidis* is usually based on surface antigens or their genes, such as the PorB and PorA (corresponding to serotype and serosubtype antigens, respectively), as well as non-cell surface components such as housekeeping enzymes or their genes using the method of multilocus enzyme electrophoresis or multilocus sequence typing (MLST), respectively [7]. The surface antigens and their genes are under constant selective pressure and therefore, resulting in greater diversity which made them suitable only for short term or localised epidemiological tracking. In contrast, housekeeping enzymes and their genes are neutral (not under selection) and are therefore, ideal targets for long term and global epidemiology [4]. In MLST, isolates with unique allelic profiles are assigned to sequence types (STs) and related STs are grouped together into clonal complexes (CCs). Most IMD, especially those occurring in clusters or epidemics are caused by strains recognised as hypervirulent clones [4]. Other additional targets may include the iron-regulated outer membrane protein FetA [8], as well as other potential non-capsule protein-based vaccine components (see below).

The capsules of meningococci are well known virulence factor by offering the pathogen resistance to innate immunity including activation of the alternative pathway of complement. Therefore, vaccines based on capsules of serogroups A, C, W, and Y (MenA, C, W, and Y) have been successfully used to control these serogroups of meningococci [9]. However, the capsule of serogroup B meningococci (MenB) is non-immunogenic due to its cross-reactivity to host tissue and therefore, development of a MenB vaccine based on its capsule is not feasible [10]. Genome sequencing of *N. meningitidis* [11] has opened a new approach, called reverse vaccinology, to vaccine development [12]. Two new MenB vaccines have been licensed in some countries. Protein-based meningococcal vaccines developed against MenB may have the potential to protect also against many clones belonging to any serogroups, including MenC, MenY, and MenW [13] or even non-capsular clones. The 4CMenB vaccine or Bexsero® (Novartis) [14] is licensed for use in Europe, Canada, the United States, and Australia, while the recombinant bi-valent vaccine Trumenba® based on factor H binding protein (fHbp), also known as lipoprotein LP2086 [15], is licensed for use in the United States. The Bexsero® vaccine is made up of three major components, fHbp, subfamily B or variant 1, peptide 1; Neisserial Heparin Binding Antigen (NHBA) peptide 2; Neisseria Adhesin A (NadA) peptide 8, combined with the outer membrane vesicle (OMV) vaccine prepared from the New Zealand MenB strain NZ98/254 which has been used for control of the MenB epidemic in New Zealand caused by the MenB strain of B:4:P1.4 (Lineage 3 or ST-41/44 CC) [16]. Meningococcal fHbp is divided into 2 subfamilies, A and B which correspond to variant 2/3 and 1, respectively [17, 18]. Proteins within a subfamily share a relatively high degree of similarity and therefore, cross-protection between peptides within a subfamily is feasible while peptides between subfamilies are more divergent and do not provide cross-protection [18]. Variants of NHBA have also been shown in animal studies to offer cross-protection [19]. NadA is made up of at least 4 variants, 1, 2/3, 4/5, and 6; with proteins within variants 1, and 2/3 shown to provide cross-protection [20]. The protection afforded by OMV vaccine is PorA or serosubtype-specific and the major immunological epitope resides on the PorA VR2 region. Thus OMV vaccine made from MenB strain NZ98/254 offers only protection against meningococcal strains expressing the PorA antigen P1.4 [16].

The epidemiology of invasive meningococcal disease in Québec, Canada, has been dominated by the ST-269 clone of MenB in the last decade associated with outbreaks or clusters of cases in certain regions of the province [21]. The aim of the current study is to document the trend since our last report as well as to expand on our previous studies to include an analysis of the genetic diversity of the newer MenB vaccine antigens, i.e. fHbp, NHBA, NadA, and PorA. This study will provide good baseline data before the newer MenB vaccines are introduced in the population.

## Methods

### Isolates of *N. meningitidis*

Individual IMD case isolates (n = 263), defined by their isolation from normally sterile body sites of individual patients were included in this study. These isolates constituted all the IMD isolates received at the National Microbiology Laboratory (NML) of the Public Health Agency of Canada between the period of 1 January 2009 and 31 December 2013.

### Identification and typing of *N meningitidis*

Isolates of *N. meningitidis* were identified at the local hospitals and their identity was confirmed and they were serogrouped at the Laboratoire de santé publique du Québec using rabbit anti-meningococcal antisera produced by the NML. Identification of serotype and serosubtype was done by a monoclonal antibody kit containing antibodies to serotypes 1, 2a, 2b, 4, 14, and 15; and serosubtypes P1.1, P1.2, P1.4, P1.5, P1.6, P1.7, P1.9, P1.10, P1.12, P1.13, P1.14, P1.15, and P1.16 (Rijksinstitut voor Volksgezondheid en Milieu, National Institute of Public Health, Bethoven, The Netherlands) using indirect whole cell ELISA [22]. Mouse monoclonal antibodies to serotype 2c, 17, 19, and serosubtype P1.19 were kind gifts from Dr. Wendell Zollinger of the Walter Reed Army Institute of Research (Maryland, U.S.A.). Clonal analysis was done by MLST [23] and ST/CC were assigned using tools available in the Neisseria MLST website (http://pubmlst.org/neisseria/). PorA genotype was determined by PCR amplification of the variable region gene fragments followed by standard DNA sequencing technique using published methods [24, 25]. The nomenclature given in the *N. meningitidis* PorA variable region database (http://pubmlst.org/neisseria/PorA/) and by de Filippis et al. [26] was followed. The *fHbp*, *nhba*, and *nadA* gene sequences were determined by PCR amplification and standard sequencing reactions following the protocols described by Lucidarme et al. [27]; and their peptide types were determined by the online tools available from the Neisseria.org website (http://pubmlst.org/neisseria). The nomenclature scheme for fHbp peptide according to Novartis was used: e.g. variant 1, peptide 1 was described as 1.1, etc.

Phylogenetic trees were constructed based on the predicted amino acid sequences of fHbp and NHBA peptides using software provided in the DNASTAR Lasergene 12.1 (Madison, Wisconsin, U.S.A.).

## Results

### Serogroup distribution per year

During the period 2009-2013, 352 IMD cases were reported to Public Health authorities in Québec. Our study included 263 individual case isolates obtained from culture-confirmed IMD patients which constituted 75 % of all IMD reported in Québec. Overall, there were 229 (87 %) MenB, 16 (6 %) MenY, 10 (4 %) MenW, 7 (3 %) MenC, and 1 isolate of MenX (Table 1). This study did not include the culture-negative and PCR diagnosed IMD cases, and this might be the major reason for the smaller number of case isolates included in this study compared to the total number of cases reported in the province.

**Table 1:** Distribution of *Neisseria meningitidis* serogroups among culture-positive invasive meningococcal disease cases in Québec, Canada, 2009-2013

	Number (%) of cases by serogroups					Total # of cases
Years	B	C	Y	W135	X	
2009	49 (90.7)	1 (1.9)	1 (1.9)	3 (5.5)	0 (0.0)	54
2010	42 (87.5)	2 (4.2)	3 (6.2)	1 (2.1)	0 (0.0)	48
2011	50 (83.3)	1 (1.7)	5 (8.3)	3 (5.0)	1 (1.7)	60
2012	41 (82.0)	3 (6.0)	5 (10.0)	1 (2.0)	0 (0.0)	50
2013	47 (92.2)	0 (0.0)	2 (3.9)	2 (3.9)	0 (0.0)	51
All years	229 (87.0)	7 (2.7)	16 (6.1)	10 (3.8)	1 (0.4)	263

### Clonal analysis of MenB

Among the 229 individual MenB case isolates, 220 or 96 % were grouped by MLST into 11 CCs, while 9 isolates did not belong to any known CC according to the current MLST typing scheme (http://pubmlst.org/neisseria/; accessed April 30, 2015). The most commonly occurring CCs were ST-269 CC (n = 159 or 69.4 %), followed by ST-41/44 CC (n = 43 or 18.8 %), ST-32 CC (n = 5 or 2.2 %), ST-35 CC (n = 4 or 1.8 %), and ST-461 CC and ST-1157 CC (each with 2 isolates or 0.9 %). There were also 5 other CCs (ST-37, ST-60, ST-103, ST-213, and ST-254 CCs) each with only a single isolate (see Additional file 1: Table S1).

Despite being the most frequently occurring CC with 159 isolates, 91 % (n = 145) of the isolates in the ST-269 CC belonged to a single ST (ST-269). There were 9 other STs with 7 of them (ST-565, ST-1986, ST-5494, ST-8767, ST-8772, ST-8924, and ST-8880) being single locus variants (SLVs) of ST-269 while ST-13 and ST-2738 were double, and triple locus variants, respectively. Most (n = 150 or 94 %) expressed the serotype antigen 17, with 8 isolates being non-serotypeable (NT), and 1 isolate of serotype 14,19. One hundred and twenty two isolates (77 %) expressed the serosubtype antigen P1.19 and 25 isolates (16 %) expressed the serosubtype antigen P1.9. There were 6 isolates that were non-serosubtypeable (P1.-), and 1 isolate each of serosubtypes P1.6; P1.7; P1.12; P1.16; P1.5,2; and P1.15,29.

In contrast to the ST-269 CC, the 43 MenB case isolates that belonged to the ST-41/44 CC were grouped into 23 different STs (Additional file 1: Table S1). Although 1 ST (ST-571) was common (responsible for 16 isolates or 37 % of the ST-41/44 CC), 19 other STs were represented by a single isolate only, and another 3 STs with multiple isolates (ST-944, n = 3; ST-282, n = 3; and ST-41, n = 2). The diversity of the ST-41/44 CC isolates was also reflected in their serotype and serosubtype antigens. Altogether, there were 7 different combinations of serotype antigens, including seventeen isolates of serotype 19; fourteen of serotype 4; four of serotype 15,19; three of serotype 17; two of serotype 1,19; and one each of serotypes 15, and 14,19. There was only one isolate that was non-serotypeable (NT). There were 8 different combinations of serosubtype antigens, including twenty of P1.9; five of P1.6; three of P1.13; two each of P1.4; P1.5; P1.12; and one each of P1.14; and P1.5, 2. Seven isolates were non-serosubtypeable (P1.-). The antigenic formula of the ST-571 isolates appeared to be generally uniform with thirteen isolates typed as B:19:P1.9; and one isolate each of B:1,19:P1.9; B:15,19:P1.9; and B:15,19:P1.-.

The other MenB case isolates (n = 27), whether belonging to a known CC or that did not belong to any known CC, were in general presenting diverse STs, serotype or serosubtype antigens. No one ST, nor serotype/serosubtype was found in large numbers.

### Clonal analysis of non-group B meningococci

All 7 MenC isolates belonged to ST-11 (ST-11 CC) and expressed the serotype antigen 2a. Three different serosubtype antigen combinations were detected including four isolates of P1.5; two isolates of P1.7,1; and one isolate of P1.2.

The four different STs found among the 10 MenW isolates all belonged to the ST-22 CC. Besides the single isolate of ST-22, there were 7 isolates of ST-184, and 1 isolate each of ST-1617 and ST-2625; with the latter 3 STs being SLVs of ST-22. All ten MenW were NT; five were positive for P1.6; one was positive for P1.14; and four were P1.-.

Twelve of the 16 MenY were grouped into 4 CCs, with ST-23 CC being the most common, made up of 6 isolates, followed by ST-167 CC with 4 isolates, and 1 isolate each of ST-174 CC and ST-22 CC. Four MenY isolates were not classified into any known CC. Half of the MenY isolates were NT; and the remaining 8 isolates presented 4 different combinations of serotype antigens including four with serotype 14,19; two with serotype 2c; and one each of serotype 19, and 15,19. Four different combinations of serosubtype antigens were found among the MenY isolates, including six isolates with P1.5; five with P1.5,2; and one each of P1.6 and P1.9. Three MenY were P1.-. Nine different combinations of serotype:serosubtype combinations were found among the 16 MenY isolates (data provided in Supplementary Table S1, see Additional file 1).

### Characteristics of non-carbohydrate protein-based vaccine targets in MenB

Similar to the clonal data of MenB isolates, a limited number of fHbp and NHBA peptide types were predicted among isolates in the ST-269 CC (Additional file 2: Table S2). There were only three fHbp peptide types identified with 152 isolates predicted to have fHbp peptide 1.15; five for peptide 2.19 and one for peptide 1.249. In one isolate, no peptide was predicted based on its *fHbp* gene sequence identified as allele 755 which contained a frame-shift mutation. The five NHBA peptide types predicted to be produced by isolates of the ST-269 CC included peptide 21 (149 isolates), peptide 770 which differed from peptide 21 by only 1 amino acid (4 isolates), peptide 6 (3 isolates), peptide 768 (2 isolates), and peptide 122 (1 isolate).

In contrast to isolates in the ST-269 CC; isolates of ST-41/44 CC were also more diverse in their *fHbp* and *nhba* gene sequences (Additional file 2: Table S2). Ten *fHbp* gene alleles together with five *nhba* gene alleles were found among isolates in the ST-41/44 CC. They predicted fHbp peptides 1.4 (n = 4), 1.14 (n = 1), 2.19 (n = 26), 2.23 (n = 2), 2.24 (n = 2), 3.30 (n = 1), 1.100 (n = 1), 1.410 (n = 4), 2.632 (n = 1), and 1.687 (n = 1); and NHBA peptides 2 (n = 6),10 (n = 4), 29 (n = 4), 47 (n = 3), 112 (n = 25), and 286 (n = 1).

The remaining MenB isolates were made up of many different STs belonging to 9 CCs as well as some that were not assigned to any known CC. Because the number of isolates in each CC or ST were very small, they would not be described individually but were grouped together for an overview of MenB strains. Of the 229 MenB IMD isolates in Québec, 25 fHbp peptide types were identified based on their *fHbp* gene sequences, including 12 peptide types with 175 isolates (76.4 %) belonging to variant 1 or subfamily B; and 13 peptide types with 52 isolates (22.7 %) belonging to subfamily A (10 peptides with 47 isolates belonged to variant 2; and 3 peptides with 5 isolates belonged to variant 3) (Table 2). In addition, there was 1 isolate with a *fHbp* gene allele 755 that did not suggest a peptide would be produced and another isolate (ST-6977) whose *fHbp* gene sequence suggested a genetic recombination, similar to that described for strain M99 241177 (GenBank JF916571) [28]. Based on their *nhba* gene sequences, 21 NHBA peptide types were expected to be present in the 229 MenB isolates in Québec. The most common NHBA peptide was peptide 21 with 154 isolates and another 4 isolates predicted to synthesise peptide 770, which was related to NHBA peptide 21 but with only 1 amino acid difference. Other common NHBA peptide types included peptide 112 (25 isolates), peptide 2 (6 isolates), and peptides 6, 10, 47, and 122, each with four isolates. The remaining NHBA peptide types were made up of either 1 or 2 isolates each (Table 3).

**Table 2:** Factor H binding protein (fHbp) peptide types in invasive *Neisseria meningitidis* from Québec, Canada, 2009-2013

Novartis variant 1	Peptides	# of isolates (serogroup)
(Pfizer subfamily B)	1.1	2 (B, two ST-32 cc)
	1.4	5 (B, four ST-41/44 cc, one ST-35 cc)
	1.12	2 (B, one UA^a^; C, one ST-11 cc)
	1.13	5 (B, one ST-60 cc, one ST-254 cc, two ST-1157 cc, one UA)
	1.14	1 (B, ST-41/44 cc)
	1.15	152 (B, 152 ST-269 cc)
	1.100	1 (B, ST-41/44 cc)
	1.110	1 (B, UA)
	1.129	1 (C, ST-11 cc)
	1.144	1 (B, ST-32 cc)
	1.218	1 (C, ST-11 cc)
	1.249	1 (B, ST-269 cc)
	1.278	1 (Y, ST-167 cc)
	1.410	4 (B, four ST-41/44 cc)
	1.687	1 (B, ST-41/44 cc)
Novartis variant 2	2.16	15 (3B, three ST-35 cc; 1C, ST-11 cc; 2Y, one ST-22 cc; one UA; 9W, nine ST-22 cc)
(Pfizer subfamily A)		
	2.19	32 (31B, five ST-269 cc; 26 ST-41/44 cc; 1×, one ST-32 cc)
	2.21	1 (Y, ST-174 cc)
	2.22	1 (B, ST-32 cc)
	2.23	6 (3B, two ST-41/44 CC; one ST-37 cc; 3Y, one ST-167 cc; two UA)
	2.24	5 (4B, two ST-41/44 cc, two UA; 1Y, ST-167 cc)
	2.25	9 (1B, ST-103 cc; 7Y, six ST-23 cc; one ST-167 cc; 1 W, ST-22 cc)
	2.102	2 (1B, ST-213 cc; 1C, ST-11 cc)
	2.106	1 (B, UA)
	2.527	1 (B, UA)
	2.629	1 (Y, UA)
	2.632	1 (B, ST-41/44 cc)
Novartis variant 3	3.30	2 (B, ST-41/44 cc, ST-32 cc)
(Pfizer subfamily A)	3.47	2 (B, two ST-461 cc)
	3.555	1 (B, UA)
No peptide	allele 669	2 (C, two ST-11 cc)
	allele 755	1 (B, ST-269 cc)
Recombination	disrupted gene	1 (B, UA)

^a^UA = not assigned to any known clonal complex by pubmlst.org/neisseria

**Table 3:** Neisserial Heparin Binding Antigen (NHBA) peptide types in invasive *Neisseria meningitidis* from Québec, Canada, 2009-2013

NHBA peptide	# of isolates (serogroup)
2	6 (B, six ST-41/44 cc)
3	2 (B, two ST-32 cc)
5	2 (B, two ST-32 cc)
6	6 (4B, three ST-269 cc; one UA^a^; 2Y; two ST-23 cc)
7	2 (Y, two ST-23 cc)
8	2 (Y, two ST-23 cc)
9	6 (Y, three ST-167 cc; three UA^a^)
10	4 (B, four ST-41/44 cc)
12	1 (B, ST-37 cc)
20	21 (2B, one ST-32 cc; one ST-254 cc; 7C, seven ST-11 cc; 10W; ten ST-22 cc; 2Y, one ST-22 cc, one UA)
21	154 (B, 149 ST-269 cc, three ST-35 cc, two UA)
24	2 (B, one ST-60 cc, one ST-103 cc)
29	6 (B, four ST-41/44 cc, one ST-213 cc, one UA)
47	4 (B, three ST-41/44 cc, one UA)
112	25 (B, 25 ST-41/44 cc)
114	2 (B, two ST-1157 cc)
122	4 (B, one ST-269 cc, three UA)
197	1 (B, ST-461 cc)
286	1 (B, ST-41/44 cc)
287	1 (B, UA)
680	1 (Y, ST-174 cc)
766	1 (B, ST-461 cc)
767	1 (X, ST-32 cc)
768	2 (B, two ST-269 cc)
769	1 (Y, ST-167 cc)
770	4 (B, four ST-269 cc)
771	1 (B, ST-35 cc)

^a^UA = not assigned to any known clonal complex by pubmlst.org/neisseria

The *nadA* gene could not be detected in MenB strains belonging to ST-269 and ST-41/44 CCs. In the other MenB strains, the *nadA* gene could only be found in isolates belonging to the ST-32 CC (n = 5); ST-1157 CC (n = 2); ST-213 CC (n = 1), and ST-336 (n = 1). However, the *nadA* genes in isolates of ST-1157 CC (allele 20) and ST-213 CC (allele 34) did not predict to produce any peptide due to frame-shift mutations. In the single isolate of ST-336, the *nadA* gene was found to predict NadA-2/3 peptide 3. Four of the 5 ST-32 CC isolates had the *nadA* gene which predicted NadA-1 peptide 1; while the *nadA* gene in the fifth isolate was predicted to encode NadA-1 peptide 85.

Among the 229 MenB case isolates, only two expressed the serosubtype antigen P1.4; with the PorA genotype of P1.7-2,4,37. Both isolates belonged to the ST-41/44 CC (one being of ST-41 and the other was ST-154).

### Characteristics of non-carbohydrate protein-based vaccine targets in non-serogroup B meningococci

In two of the seven MenC isolates, their f*Hbp* gene allele 669 contained a frame-shift mutation that led to a premature stop codon and therefore, no fHbp peptide would be expected in them. The other five isolates each have a different *fHbp* gene allele predicting the following fHbp peptides (1.12, 2.16, 2.102, 1.129, and 1.218); three of which belonged to variant 1 and two to variant 2. The *nadA* genes (allele 29) in four MenC isolates were disrupted by presence of an IS*1301* element, and therefore, no peptide were expected in them. In one MenC isolate, its *nadA* gene predicted a NadA-2/3 peptide 2; and in two MenC isolates, their *nadA* gene predicted a NadA-2/3 peptide 3. All seven MenC isolates were found to have *nhba* genes that predicted a NHBA peptide 20.

Of the 16 MenY isolates, seven fHbp peptide types were predicted based on their *fHbp* gene sequences. Other than the one isolate that was predicted to produce a variant 1 fHbp peptide 1.278, the remaining 15 MenY isolates were predicted to encode variant 2 fHbp peptides 2.16, 2.21, 2.23, 2.24, 2.25, and 2.629; with peptide 2.25 being the most common peptide found in seven isolates. Seven NHBA peptide types (6, 7, 8, 9, 20, 680, and 769) were identified based on their *nhba* gene sequences. Peptide 680 was related to peptide 6 but with 1 amino acid difference. Only the single ST-1466 (ST-174 CC) MenY was found to have a *nadA* gene allele 80, which predicted a NadA-2/3 peptide 8. The *nadA* gene was not detected in all the other MenY isolates.

All ten MenW isolates were found to have *fHbp* genes that encoded for variant 2 peptides, with nine of them predicted to encode for peptide 2.16, and only one isolate was predicted to produce a fHbp peptide 2.25. All ten MenW were found to have *nhba* gene allele encoding for NHBA peptide 20. None of the MenW was found to harbor the *nadA* gene and therefore, no NadA protein was expected to be present in these MenW isolates.

The single MenX was found to have *fHbp* and *nhba* genes to encode for fHbp peptide 2.19 (variant 2), and NHBA peptide 767. No *nadA* gene was detected in the MenX isolate.

### Predicted coverage by the 4CMenB vaccine based on vaccine target gene sequence analysis

There were altogether 30 fHbp peptide types identified among the invasive *N. meningitidis* strains found in Québec. Their frequencies of occurrence in different serogroups are described in Table 2. In addition, there were 3 isolates with *fHbp* gene mutations that would prevent synthesis of a mature fHbp peptide. In another isolate, there was a recombination resulting in an interruption of its *fHbp* gene by a foreign unrelated gene (GenBank JF916571) [28]. There were 27 different NHBA peptide types and their distribution according to serogroup is described in Table 3.

For MenB isolates, none was found to have more than two vaccine antigens that matched exactly with the 4CMenB components and only two isolates were found to have PorA P1.4 and NHBA peptide 2 that matched exactly with the respective components found in the vaccine. Another six MenB isolates were found to have one antigen, either fHbp peptide 1.1 (two isolates) or NHBA peptide 2 (four isolates), that matched exactly with the corresponding component found in the 4CMenB vaccine (Table 4). Assuming all isolates with fHbp variant 1 and NadA variants 1 and 2/3 peptides would be covered by the vaccine, two MenB isolates might be regarded to be covered by three components of the vaccine by virtue of their PorA P1.4, fHbp peptide 1.4, and NHBA peptide 2 antigens; and five MenB isolates might be regarded to be covered by two components of the vaccine by virtue of their variant 1 fHbp peptides and NHBA peptide 2 (four isolates), and variant 1 fHbp peptide and NadA peptide 85 (one isolate). Another 166 MenB isolates were considered to be covered by one component of the vaccine: variant 1 fHbp (163 isolates) or NadA variants 1, 2/3 peptides (3 isolates).

**Table 4:** Invasive *Neisseria meningitidis* from Québec, Canada in 2009-2013: predicted vaccine coverage based on at least one antigen showing an exact match to the 4CMenB vaccine components or with Novartis variant 1 factor H binding protein (fHbp)/Neisseria adhesin A (NadA) peptides belonging to variants 1 to 3

				Predicted coverage based on exact match to 4CMenB components^a^			
CC	ST	Serogroup	# of isolates	fHbp^b^	NHBA	NadA-2/3^c^	PorA
ST-41/44	ST-41	B	1	Possibly, 4	Yes	No	Yes
	ST-154	B	1	Possibly, 4	Yes	No	Yes
	ST-41	B	1	Possibly, 4	Yes	No	No
	ST-1194	B	1	Possibly, 4	Yes	No	No
	ST-8052	B	1	Possibly, 14	Yes	No	No
	ST-10619	B	1	Possibly, 100	Yes	No	No
ST-32 CC	ST-32	B	2	Yes, 1	Unknown	No	No
ST-174 CC	ST-1466	Y	1	No	Unknown	8	No
Additional coverage based on strains predicted to express NadA-1,2/3 and/or Novartis variant 1 fHbp peptides							
ST-269 CC	ST-269	B	143	Possibly, 15	Unknown	No	No
	ST-1986	B	4	Possibly, 15	Unknown	No	No
	ST-5494	B	3	Possibly, 15	Unknown	No	No
	ST-8767	B	1	Possibly, 15	Unknown	No	No
	ST-8880	B	1	Possibly, 15	Unknown	No	No
	ST-2738	B	1	Possibly, 249	Unknown	No	No
ST-32 CC	ST-33	B	1	Possibly, 144	Unknown	85	No
	ST-2726	B	2	No	Unknown	1	No
ST-35 CC	ST-278	B	1	Possibly, 4	Unknown	No	No
ST-1157 CC	ST-1157	B	2	Possibly, 13	Unknown	No	No
ST-60 CC	ST-10587	B	1	Possibly, 13	Unknown	No	No
ST-254 CC	ST-10332	B	1	Possibly, 13	Unknown	No	No
Not assigned^d^	ST-336	B	1	No	Unknown	3	No
	ST-7655	B	1	Possibly, 13	Unknown	No	No
	ST-7704	B	1	Possibly, 110	Unknown	No	No
	ST-9248	B	1	Possibly, 12	Unknown	No	No
ST-11 CC	ST-11	C	2	No	Unknown	3	No
	ST-11	C	1	No	Unknown	2	No
	ST-11	C	1	Possibly, 12	Unknown	No	No
	ST-11	C	1	Possibly, 129	Unknown	No	No
	ST-11	C	1	Possibly, 218	Unknown	No	No
ST-167 CC	ST-6171	Y	1	Possibly, 278	Unknown	No	No

^a^4CMenB vaccine components include: factor H binding protein (fHbp) peptide 1; Neisseria Heparin Binding Antigen (NHBA) peptide 2; Neisseria adhesion A (NadA)-2/3 peptide 8; and outer membrane protein porin A (PorA) P1.4

^b^Novartis variant 1 peptides were indicated by their peptide number possibly covered by the vaccine; whereas variants 2/3 peptides were labelled as No to indicate no match or not covered by the 4CMenB vaccine

^c^NadA variant 1-3 peptides were indicated by their peptide number; whereas No was used to indicate absence of the *nadA* gene

^d^Not assigned to any known clonal complex according to the current Neisseria MLST website (http://pubmlst.org/neisseria/)

For the non-serogroup B IMD strains in Québec, none was found to have *fHbp* and *nhba* genes encoding for fHbp peptide 1.1 and NHBA peptide 2. However 1 MenY isolate (ST-1466) was found to possess a *nadA* gene for NadA peptide 8 found in the 4CMenB vaccine. Another 4 isolates (3 MenC and 1 MenY) were found to have *fHbp* genes that predicted variant 1 fHbp peptides. Also 3 MenC were found to have *nadA* genes encoding for NadA-2/3 peptides, likely to have enough similarity with the NadA peptide component in the 4CMenB to be regarded as covered by the vaccine.

Regardless of serogroup, a total of 9 isolates could be regarded to be covered by the 4CMenB vaccine by possession of at least 1 antigen showing an exact match to the vaccine components; and an additional 172 isolates might be covered by virtue of possession of at least 1 antigen showing a partial match with the vaccine components, e.g. fHbp variant 1 peptides.

**Fig. 1:**
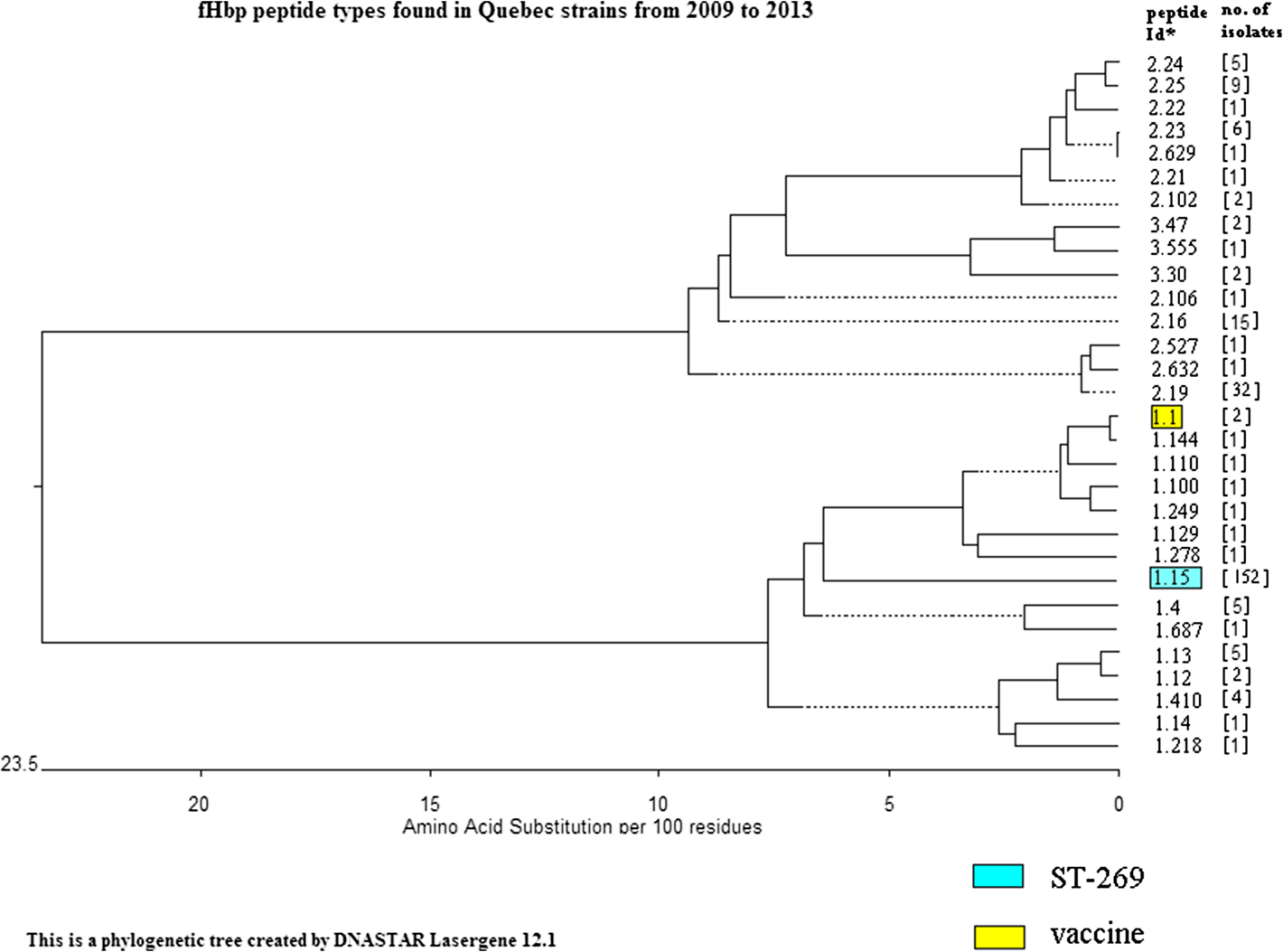
Phylogenetic tree to show the relationship of factor H binding protein peptide types identified in invasive *Neisseria meningitidis* from Québec, Canada, 2009-2013. *Peptide Id from the Neisseria.org website (http://pubmlst.org/neisseria/fHbp/). Factor H binding protein peptide Id 1.1, 1.4, 1.12, 1.13, 1.14, 1.15, 1.100, 1.110, 1.129, 1.144, 1.218, 1.249, 1.278, 1.410, and 1.687 belong to Novartis variant 1 or subfamily B. Factor H binding protein peptide Id 2.16, 2.19, 2.21, 2.22, 2.23, 2.24, 2.25, 2.102, 2.106, 2.527, 2.629, and 2.632 belong to Novartis variant 2 or subfamily A. Factor H binding protein peptide Id 3.30, 3.47, and 3.555 belong to Novartis variant 3 or subfamily A. Dotted line indicates a negative branch length

**Fig. 2:**
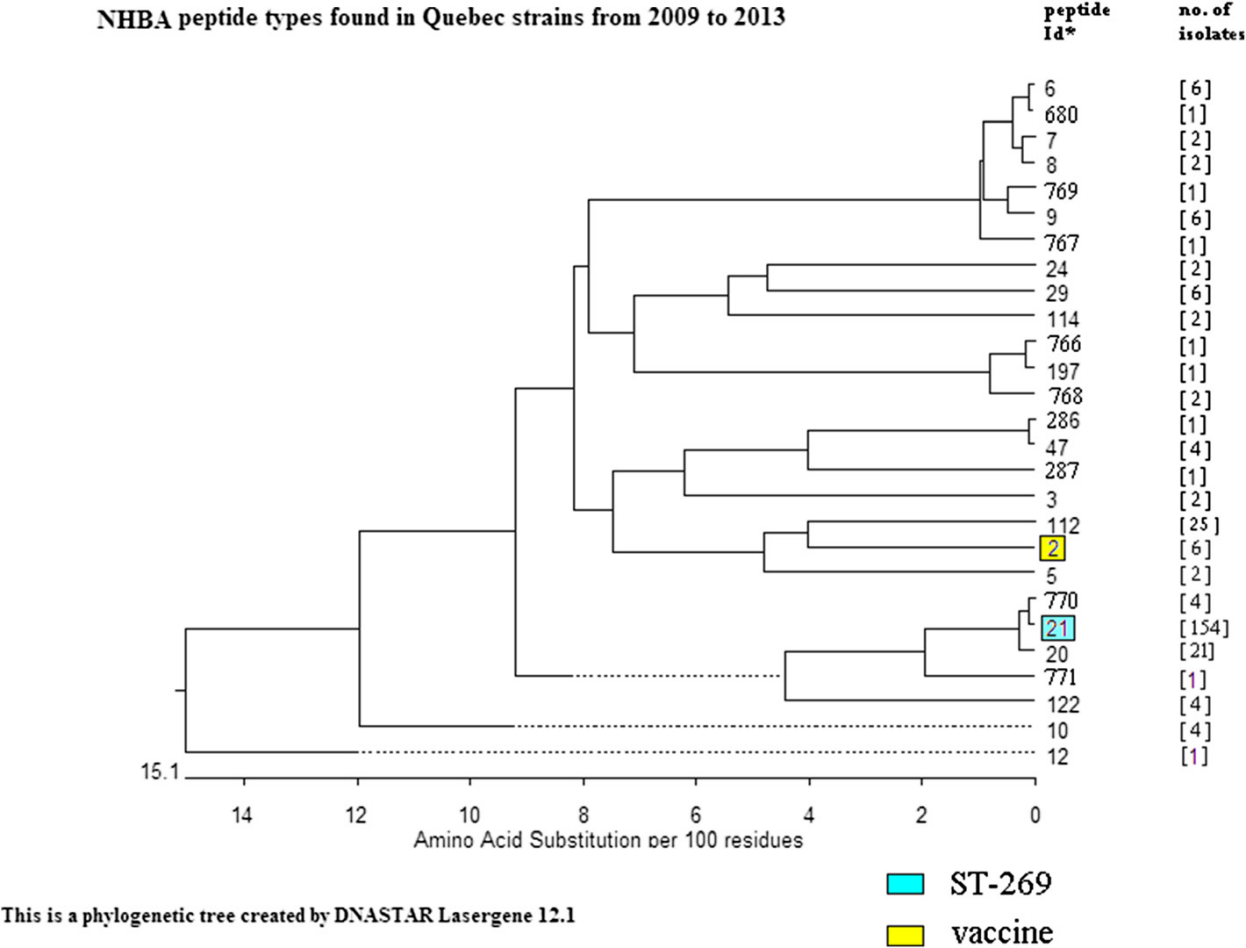
Phylogenetic tree to show the relationship of Neisseria Heparin Binding Antigen peptide types in invasive *Neisseria meningitidis* from Québec, Canada, 2009-2013. *Peptide Id from the Neisseria.org website (http://pubmlst.org/neisseria/NHBA). Variants were labelled with the peptide Id. Dotted line indicates a negative branch length

The diversity of the fHbp and NHBA peptides found in the invasive *N. meningitidis* strains in Québec are presented in Figs. 1 and 2, respectively. With respect to fHbp peptides, even among the variant 1 peptides, considerable amino acid diversity was found among IMD isolates in Québec (Fig. 1; bottom half of the figure). In addition, it was evident that peptides of the variants 2 and 3 were more distantly related to peptides that belong to variant 1. Similarly, NHBA peptides found in the Québec IMD isolates were also grouped into clusters, with some clusters of peptides showing various degree of diversity with the vaccine peptide 2 (Fig. 2).

## Discussion

Factor H binding protein (fHbp) is one of *Neisseria meningitidis* virulence factors and is found in two meningococcal vaccines, Bexsero® and Trumenbal®. Characterization of fHbp from various *N. meningitidis* strains have identified 2 subfamilies (A and B) or 3 variants (variants 1, 2, 3) of fHbp. Subfamily A fHbp is equivalent to variants 2 and 3 while subfamily B fHbp is equivalent to variant 1. Immunological cross-reactivity and cross-protection between different fHbp within a subfamily have been observed but this is not extended to fHbp from different subfamilies [18]. Bexsero® also known as 4CMenB, is made up of multi-components with fHbp being only one of them. The fHbp present in this vaccine is restricted to peptide 1.1 from subfamily B, which presumably will cover strains expressing subfamily B fHbp. Broader protective nature of Bexsero® is extended by its other components, including NHBA, NadA and PorA P1.4. On the other hand, Trumenbal® is composed of only fHbp but it is a bivalent fHbp vaccine with 2 peptides present representing both subfamily A and subfamily B that allows it to cover strains expressing either subfamily of fHbp. This study provided a comprehensive genetic analysis of this virulence factor found in invasive *N. meningitidis* strains in the province of Quebec, Canada over the five years period of 2009-2013, just before the licensure of either of these vaccines.

This study was based on our previous reports of a changing epidemiology of IMD in the province of Quebec [21, 29, 30] but the focus was on characteristics of invasive *N. meningitidis* strains and the genetic diversity of vaccine antigens found in the newer MenB vaccines. Results of this study documented the continued dominance of MenB as a cause of IMD in Québec, with 92 % of the IMD case isolates in 2013 belonging to this serogroup (Table 1), and in particular the ST-269 CC. Compared to the study that examined IMD case isolates in Québec from 2003 to 2010 which consisted of 180 MenB isolates belonging to the ST-269 CC with 91.7 % due to a strain typed as ST-269 [30], the present study showed that the % of ST-269 isolates in the ST-269 CC has not changed and remained as 91.2 % (145 of 159 isolates of MenB ST-269 CC). In the original report of the emergence of the ST-269 clone, there were only 3 STs identified among the MenB isolates in this CC in Québec [29]. Then the number of STs within this CC expanded to involve 11 STs [30], with 8 STs newly identified when compared to the previous finding [29]. The present study also identified ten STs within the Quebec ST-269 CC but four of the STs found previously [30] were not found in the present study, and three new STs not previously found were included in here. Also of interest is ST-275 and its SLV ST-1163 found in the period of 2003-2010 did not appear to have expanded to cause IMD in Québec. This was in contrast to the endemic MenB disease reported in England/Wales, where the ST-275 clone has expanded while ST-269 clone has decreased in frequency of detection among IMD case isolates [27]. The difference between the molecular epidemiology of MenB disease in Québec and England/Wales might be in Québec MenB ST-269 has been associated with clusters of cases whereas in England/Wales, the MenB disease has been characterized as endemic disease without any single clone dominating. Our data may suggest that ST-269 strain in Québec might have a unique advantage in establishing itself in the population to cause significant disease over an extended period of time [30]. Indeed we speculated that MenB strain of ST-269 has the potential to cause outbreaks [31].

Among the MenB ST-41/44 CC isolates, ST-571 continued to be a common ST found, responsible for 37 % of the MenB in this CC, similar to the finding reported previously [30]. Isolates of ST-571 also appeared to have a uniform antigenic formula expressing serotype antigen 19 (in all 16 isolates), and serosubtype antigen P1.9 (in 15 isolates) together with the PorA genotype of P1.18-7, 9, 35-1 (in 15 isolates). Other MenB isolates within the ST-41/44 CC or other CCs or those not belonging to any known CC can essentially be described as diverse, not restricted to any particular ST or antigenic form.

Analysis of the 4CMenB vaccine antigen genes among the various meningococcal serogroups and CCs have provided some interesting observations (Table 4). Although MenB that belonged to the ST-269 CC might not have any antigens that matched exactly with the 4CMenB vaccine components, a significant percentage (96 % or 153 out of 159 isolates) were predicted to be covered based on the majority of them possessed *fHbp* genes that encoded for variant 1 fHbp peptide 1.15. The coverage of this clone by the vaccine was further supported by an in vitro assay Meningococcal Antigen Typing System (MATS) that measures expression, and cross-reactivity of the vaccine antigens on clinical isolates. Using MATS, the majority of strains within the ST-269 CC were covered by fHbp and NHBA; and the coverage for strains typed as ST-269 was 95 % [32]. Also all five MenB isolates of the ST-32 CC were also predicted to be covered by the vaccine, either because they possessed genes that encoded for a matching 4CMenB vaccine component (variant 1 fHbp peptide 1.1; two isolates) or with genes that encoded for NadA variants 2/3 peptides (3 isolates). This finding is in line with other observations that many MenB of ST-32 CC are likely to be covered by the 4CMenB vaccine [19]. In contrast a small number of MenB isolates of the ST-41/44 CC (14 % or 6 out of 43 isolates) were predicted to be covered, possibly because of the heterogeneity of strains found in this CC. Of the 27 MenB that did not belong to ST-269 and ST-41/44 CCs, only 12 or 44 % were predicted to be covered by the 4CMenB vaccine. Therefore, aside from ST-269, ST-32 and ST-41/44 CCs, MenB of other clonal types in Quebec might be only poorly covered by the vaccine according to the strains analysed in this study. Besides MenB, 6 of the 7 ST-11 MenC were predicted to be covered by the 4CMenB vaccine by virtue of either their *nadA* genes encoding for variant 2/3 NadA peptides (3 isolates) or *fHbp* genes encoding for variant 1 fHbp (3 isolates). In contrast, none of the 10 MenW and only 2 of the 16 MenY were predicted to be covered by the vaccine (Table 4).

The number of MenB isolates in this study predicted to synthesize variant 1 fHbp was greatly influenced by the presence of a large number of the ST-269 CC isolates which predominantly predicted to have fHbp peptide 15 (variant 1). If this clone was removed from the calculation, the number and percentage of MenB isolates predicted to have variant 1, 2, 3 fHbp would be 23, 47, and 5 isolates or 30.7, 62.7, and 6.7 %, respectively. The percentage of MenB in Québec without the ST-269 CC would be more comparable to the percentage of MenB in Western Canada predicted to have variant 1 fHbp (37.7 %) [33]. In Western Canada, the percentage of MenB predicted to encode for variants 2 and 3 fHbp were 34.8 % and 27.5 %, respectively The higher percentage of variant 3 fHbp found among MenB in Western Canada (27.5 %) was due to the presence of the ST-213 cc, which were predominantly (92 %) predicted to have fHbp peptide 45 (variant 3) [33].

The number of MenC, Y, W, and X were relatively small and therefore, prediction of how well the vaccine may or may not work against these serogroups of meningococi in Québec may change when more isolates are tested. The 34 MenC, Y, W, and X isolates were found to possess diverse alleles of *fHbp* and *nhba* genes predicted to encode for 13 different fHbp and eight NHBA peptide types. However, only 4 (3 MenC and 1 MenY) isolates were predicted to have variant 1 fHbp while 28 isolates were predicted to have variant 2 fHbp; and 2 MenC were predicted not to produce fHbp due to a frame-shift mutation in their *fHbp* genes [28]. Among MenC, no two isolates were found to have identical *fHbp* gene alleles; a phenomenon linked to the ET-15 variant of MenC in the ST-11 CC [34]. Also 4 of the 7 MenC isolates were found to have their *nadA* genes disrupted by presence of IS1301 in the coding region, thereby leading to no NadA protein predicted in these isolates [35]. NadA-2/3 peptides were predicted in 3 of the 34 non-B meningococci, including 2 MenC (NadA peptide 3) and one MenY (NadA peptide 8).

Despite conjugated MenC vaccination programs implemented since 2001-2002 to cover all infants and adolescents in the province, it appears invasive MenC disease still occurred at a low level in the adult population. Six of the seven MenC cases were in the age range of 30 to 83 years (with a median age of 59.5 years); and in one case the age information is not available. Also the responsible MenC strain appears to be identical to the hyper-virulent ET-15 clone prominent in prevalence before the MenC conjugate vaccine was implemented. The continuing circulation of this MenC ET-15 clone with the potential to cause outbreaks with severe outcome [36], justifies the existence of an immunisation program consisting of two MenC conjugate vaccine doses offered at 12 months and 12 years of age, respectively [37], in order to maintain the immunity in the population and to keep the level of invasive MenC disease to a minimum in the province. Genetic analysis of new MenB vaccine targets suggested that the MenC ET-15 clone may be covered by the fact that they all have *nhba* genes for NHBA peptide 20 (which may cross-react with NHBA peptide 2), or either presence of *fHbp* genes for variant 1 fHbp peptides in three isolates or *nadA* genes that encoded for NadA peptide 2 or 3 in another three isolates.

With the implementation of the Bexsero® vaccine in regions of Quebec with the highest incidence of IMD due to the ST-269 clone (authors’ unpublished data), attention are now being focused on gathering data that would provide information on the efficacy of the vaccine, its safety profile in the field, its acceptability by the population, its effect on potential carriage and herd immunity, as well as its potential effect on the circulating population of *N. menngitidis* strains. This post-implementation surveillance data would help to improve our knowledge of this new vaccine and may assist in refining the dosage and/or schedule of the vaccine, as well as its true strain coverage.

## Conclusion

This study provided an overall molecular analysis of the invasive *N. meningitidis* in Québec in recent years with an intent to examine the genetic diversity and distribution of the newer meningococcal vaccine targets. For MenB, eight strains were predicted to have one (six isolates) or two (two isolates) antigens that matched exactly with the respective component in the 4CMenB vaccine. Six isolates (including two that also expressed the PorA P1.4 antigen) were predicted to encode NHBA peptide 2, and two isolates predicted to encode variant 1 fHbp peptide 1.1. Another 165 MenB isolates might be regarded to be covered by the vaccine by virtue of either possessing genes for variant 1 fHbp or NadA variants 1, 2/3 peptides. For the 34 non-MenB isolates, only one MenY was found to possess a *nadA* gene for the NadA peptide 8 found in the 4CMenB vaccine. In addition seven other isolates predicted to be potentially covered by the vaccine by virtue of either possession of a *fHbp* gene for variant 1 fHbp peptide (four isolates) or a *nadA* gene for variant 2/3 NadA peptides (three isolates).

The major limitation is we did not perform assay to examine expression of the newer vaccine targets in our clinical isolates and their cross-reactivities with those found in the vaccine. Also the number of isolates as a whole were small which might affect the confidence level of the finding. Furthermore, we did not have clinical data on hand to compare the virulence of the different genotypes identified, in particular those in the ST-269 CC. Therefore, further prospective studies employing these tools and approaches may allow for a more in-depth analysis of IMD in Québec, Canada.

## Additional files

Additional file 1: Table S1. A list of invasive meningococcal disease isolates in Quebec, Canada from 2009 to 2013. Additional file 2: Table S2. 4CMenB protein antigens^Δ^ predicted in serogroup B *Neisseria meningitidis* belonging to ST-269 and ST-41/44 claonal complexes (CCs) *and* isolated in Québec, Canada, 2009-2013. These additional files as well as Figs. 1 and 2 showing phylogenetic trees of factor H binding protein and Neisserial Heparin Binding Antigen peptide types were also deposited in Dryad (http://datadryad.org/).
