# Supplementary material for: Characterization of invasive Neisseria meningitidis strains from Québec, Canada, during a period of increased serogroup B disease, 2009-2013: phenotyping and genotyping with special emphasis on the non-carbohydrate protein vaccine targets
Source: BMC Microbiol. 2015 Jul 25;15:143. doi: 10.1186/s12866-015-0469-6 (PMC4514445; doi:10.1186/s12866-015-0469-6)
Supplement: Additional file 2: Table S2. — 4CMenB protein antigensΔ predicted in serogroup B Neisseria meningitidis belonging to ST-269 and ST-41/44 claonal complexes (CCs) and isolated in Québec, Canada, 2009-2013. These additional files as well as Figs. 1 and 2 showing phylogenetic trees of factor H binding protein and Neisserial Heparin Binding Antigen peptide types were also deposited in Dryad (http://datadryad.org/). [file 12866_2015_469_MOESM2_ESM.docx]

**Additional file 2: Table S2. 4CMenB protein antigens^Δ^ predicted in serogroup B *Neisseria meningitidis* belonging to ST-269 and ST-41/44 claonal complexes (CCs) *and* isolated in Québec, Canada, 2009-2013.**

Clonal complex Sequence Type fHbp peptide NHBA peptide NadA peptide PorA P1.4 # of isolates

ST-269 CC ST-269 15 21 Absent No 136

15 770 Absent No 4

15 768 Absent No 2

15 20 Absent No 1

19 6 Absent No 1

19 21 Absent No 1

Other STs 15 21 Absent No 9

19 6 Absent No 2

19 21 Absent No 1

249 122 Absent No 1

Allele 755**^#^** 21 Absent NO 1

ST-4144 CC ST-571 19 112 Absent No 11

410 112 Absent No 3

632 112 Absent No 1

687 112 Absent No 1

Other STs 19 112 Absent No 8

19 47 Absent No 3

19 286 Absent No 1

19 10 Absent No 1

19 29 Absent No 2

4 2 Absent Yes 2

4 2 Absent No 2

Clonal complex (CC) Sequence Type (ST) fHbp peptide NHBA peptide NadA peptide PorA P1.4 # of isolates

ST-41/44 CC Other STs 14 2 Absent No 1

23 10 Absent No 1

24 10 Absent No 2

23 29 Absent No 1

30 29 Absent No 1

100 2 Absent No 1

410 112 Absent No 1

**^Δ^** 4CMenB protein antigens: fHbp = factor H binding protein; NHBA = Neisserial heparin binding antigen; NadA = Neisseria adhesion A

**^#^** *fHbp* allele 755 containing a frame-shift mutation
